# Revealing the prognostic and clinicopathological significance of systemic immune-inflammation index in patients with different stage prostate cancer: A systematic review and meta-analysis

**DOI:** 10.3389/fmed.2022.1052943

**Published:** 2022-10-31

**Authors:** Wenqiang Qi, Yongheng Zhou, Zhifeng Liu, Jian Wang, Guangda Lv, Minglei Zhong, Wenfu Wang, Rongyang Li, Shouzhen Chen, Benkang Shi, Yaofeng Zhu

**Affiliations:** ^1^Department of Urology, Qilu Hospital of Shandong University, Jinan, China; ^2^Department of Urology, Taian City Central Hospital, Taian, China; ^3^Department of Urology, People's Hospital of Laoling, Dezhou, China; ^4^Department of Thoracic Surgery, Qilu Hospital of Shandong University, Jinan, China

**Keywords:** systemic immune-inflammation index, prognosis, systematic review, meta-analysis, metastatic castration-resistant prostate cancer (mCRPC), non-metastatic prostate cancer

## Abstract

**Background:**

A novel inflammatory marker called the systemic immune-inflammation index (SII) was applied to predict the prognosis of different cancers. However, the role of SII in prostate cancer (PCa) remains unclear. This systematic review aims to explore the prognostic role of SII in different stage PCa.

**Methods:**

We comprehensively searched three public databases: PubMed, EMBASE, and the Cochrane Library. The hazard ratios (HRs) and odds ratios (ORs) with 95% confidence intervals (CIs) were extracted to evaluate the association between SII and the prognosis and clinicopathological characteristics in different stage PCa patients.

**Results:**

Ten studies and 7,986 patients were enrolled in our meta-analysis, 1,442 patients were diagnosed with metastatic-castration resistant prostate cancer (mCRPC), and 6544 patients were diagnosed with non-metastatic prostate cancer (nmPCa). According to the pooled results, we found that a high SII was associated with worse overall survival (OS) in mCRPC patients (HR = 1.94, 95% CI: 1.26–3.01, *p* = 0.003), and a high SII was associated with biochemical recurrence-free survival (BFS) in nmPCa patients (HR = 1.85, 95% CI: 1.06–3.24, *p* = 0.031). But there was no significant association observed between SII and progression-free survival (PFS) in mCRPC patients (HR = 1.90, 95% CI: 0.87–4.14, *p* = 0.107). And we found that the high SII was associated with advanced tumor stage of PCa (OR = 2.19, 95% CI: 1.11–4.33, *p* = 0.024), presence of lymph node involvement (OR = 2.72, 95% CI: 1.96–3.76, *p* < 0.001) and Gleason score (OR = 1.27, 95% CI: 1.13–1.44, *p* < 0.001).

**Conclusion:**

High SII was associated with bad OS in mCRPC patients, and associated with bad BFS and some adverse pathological features in nmPCa patients. We think SII can be a prognostic predictor for PCa patients. The application of SII will advance the diagnosis and treatment of different stage prostate cancer.

## Introduction

Prostate cancer (PCa) is the second most frequent cancer, and there are 1.4 million new cases and 375,000 deaths worldwide in 2020 (1). Localized non-metastatic PCa (nmPCa) is the early stage, and most patients are in this stage at the time of diagnosis. Radical prostatectomy (RP) is the optimized option for localized disease. Approximately 45–63% of patients with localized PCa experience biochemical recurrence (BCR) after surgery within 5 years (2). So, the indicator of BCR is often described for patients' prognosis. With evolvement of the screening of prostate-specific antigen (PSA) and the management of PCa, there is an increase from 3.9 to 8.2% in the proportion of PCa diagnosed at a distant stage (3). It is estimated that approximately 700,000 out of 10 million patients who are diagnosed with PCa will develop into metastatic disease (4, 5). Chemotherapy, radiotherapy, and hormone therapy are considered as treatment options for metastatic disease (6, 7). For metastatic PCa, people are more concerned about overall survival (OS) and disease progression. In addition, some tumors will eventually progress to metastatic castration-resistant prostate cancer (mCRPC).

To predict the OS in mCRPC patients, Gleason score (GS), pathological stage (pT) and Eastern Cooperative Oncology Group (ECOG) performance status are used as independent predictors. However, these factors do not function as dynamic detecting indicators of OS in the subsequent treatment. In order to monitor the OS of mCRPC patients in follow-up treatment, some scholars have used blood inflammatory markers, such as neutrophil-to-lymphocyte ratio (NLR) and platelet-lymphocyte ratio (PLR), to improve the predictive accuracy, which indicates that higher NLR and PLR are associated with worse OS (8–10).

Due to the complexity of the immune microenvironment, the integration of the only two types of immune cells is inadequate for predicting the prognosis of PCa patients (11). A novel inflammatory marker called the systemic immune-inflammation index (SII) was first applied to predict the prognosis of PCa patients in 2016, though it has been used to predict occurrence and progression of other cancers (12–14). Fan et al. found that the ability of SII to predict the OS and radiographic progression-free survival (rPFS) of PCa patients is more powerful than NLR (15). However, the poor ability of SII to predict recurrence of PCa has shown in several other studies (16).

The predicting significance of SII in PCa remains controversial, and there is no separate meta-analysis to study its ability to predict the prognosis of PCa patients. Therefore, this study aims to explore the prognostic role of SII in PCa, and the correlation between SII and clinicopathological features of PCa.

## Methods

This systematic review and meta-analysis was conducted and reported on the basis of the Meta-Analysis of Observational Studies in Epidemiology (MOOSE) guidelines (17) and the PRISMA statement (18). The protocol of this review has been registered on the INPLASY website (https://inplasy.com/inplasy-2022-8-0014) and the number is INPLASY202280014.

### Databases and search strategies

We searched three databases until July 3, 2022, including PubMed, EMBASE, and the Cochrane Library. The literature search was conducted using the following terms: (prostate cancer OR prostate neoplasms OR prostate tumor OR prostate malignancy) AND (systemic-immune-inflammation index OR SII OR neutrophil × platelets / lymphocyte). We also searched the references of relevant reviews and meta-analyses to avoid omissions. The detailed search strategies are shown in Supplementary Table 1. The articles were independently reviewed by two reviewers (Wenqiang Qi and Yongheng Zhou) and any discrepancies were resolved by discussion with the third reviewer (Minglei Zhong).

### Selection criteria

The inclusion criteria were as follows: (I) patients were diagnosed with different stage PCa; (II) hazard ratios (HRs) and corresponding 95% confidence intervals (CIs) for pretreatment SII and survival outcomes were reported; (III) the relationships between SII and clinicopathological characteristics of PCa were reported; (IV) the cutoff value of SII was described.

The exclusion criteria were as follows: (I) ineligible article types, such as case reports, reviews and conference abstracts; (II) results of interest in the article are not available.

### Endpoints and outcome measures

The primary outcomes were as follows: (I) the predictive effect of SII on OS and PFS for mCRPC; (II) the predictive effect of SII on BFS for nmPCa.

The secondary outcomes were the association between the SII and adverse clinicopathology, including pathological tumor stage ≥3 (pT ≥3), Gleason score GS ≥7, pathological lymph node stage ≥1 (pN ≥1) and positive surgical margins.

### Data extraction and quality assessment

Two reviewers (Wenqiang Qi and Yongheng Zhou) independently performed literature reading and extracted the corresponding data using a predefined table. All inconsistencies were resolved through discussions. The following data were extracted from each study: (I) publication data: the name of first author, year of publication, age, country, study design and study period; (II) experimental data: tumor type, the option of treatment, follow-up time, sample size of participants and the cutoff value of SII; (III) outcome data: OS, BFS, PFS. Furthermore, all outcome variables were extracted in the form of HRs with 95% CIs. The quality of the included cohort studies was evaluated using the Newcastle-Ottawa Quality Assessment Scale (NOS) (19). Studies with NOS score equal to or higher than 7 were eligible for our meta-analysis.

### Statistical analysis

We calculated the HRs with 95% CIs to evaluate the associations between SII and survival outcomes. The heterogeneity level was quantified using Cochran's Q Test and *I*^2^ statistics. *I*^2^ of 25%, 50%, and 75% represent low, moderate, and considerable heterogeneity, respectively. If there is low or medium heterogeneity, fixed effects models were employed to estimate pooled effect sizes. And if there is considerable heterogeneity, random effects models were employed to estimate pooled effect sizes in order to reduce possible bias. In addition, sensitivity analyses were conducted by omitting individual studies sequentially to detect their impact on the overall estimates. The pooled odds ratios (ORs) and 95% CIs were performed to detect the association between SII and clinicopathological factors. The statistical significance was defined as a 2-sided *P*-value < 0.05. All data processing and statistical analyses were conducted using STATA (version 14; StataCorp LLC, University of Texas Station, USA).

## Results

### Literature search

**Figure 1:**
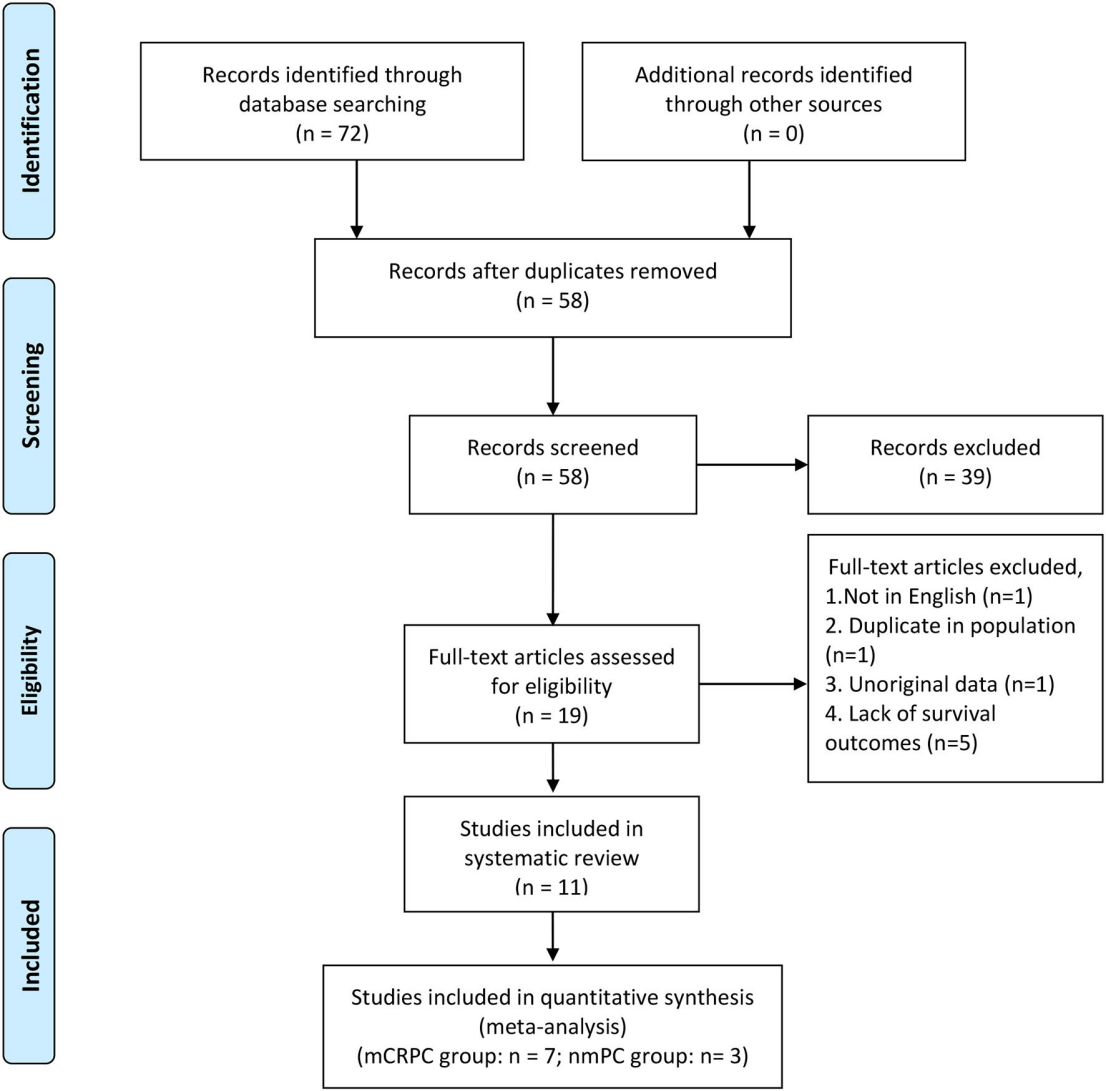
PRISMA flow diagram of literature retrieval. PRISMA, Preferred Reporting Items for Systematic Reviews and Meta-Analyses.

We identified a total of 72 records from the initial database search, of which 49 studies were from PubMed database, 21 studies were from EMBASE database and 2 studies were from the Cochrane library database. After excluding duplications and screening the full-texts, 11 studies (12, 15, 16, 20–27) were included in this systematic review, and 10 (12, 15, 20–27) of which were enrolled in the meta-analysis. Patients in 7 studies (12, 15, 20–23, 26) were diagnosed with mCRPC, and those in the other 3 studies (24, 25, 27) were diagnosed with nmPCa. One study (16) was excluded from the meta-analysis because it didn't provide the specific value of HR and 95% CI. A flow diagram outlining of the study selection process is presented in Figure 1.

### Characteristics of the included studies and patients

We divided patients into the mCRPC group and nmPCa group based on the diagnosis. And the baseline characteristics and major survival outcomes of each study are summarized in Tables 1, 2. There are 9 retrospective cohort studies (12, 15, 20, 22–27) and one prospective study (21) included in the meta-analysis. Four studies (15, 22, 23, 27) were conducted in Asia, and 6 studies (12, 20, 21, 24–26) were conducted in Europe. These studies were published between 2016 and 2022 with a sample size between 80 and 6,039. And a total of 7,986 patients were enrolled in our meta-analysis.

**Table 1:** Baseline characteristics of included mCRPC patients.

**References**	**Country**	**Study design**	**Study period**	**Tumor type**	**Treatment**	**Sample size**	**Age**	**Cutoff value**	**Outcome**	**Follow-up (months)**	**NOS SCORE**
Bauckneht et al. (20)	Italy	RCS	2013–2020	mCRPC	[^223^Ra] RaCl_2_ + ADT	519	74 (50–90)	768.8	OS	10.7	8
Donate-Moreno et al. (21)	Spain	PCS	Until 2018.12	mCRPC	AA/EZ	80	72.7	535	OS	19 [0.43–77.1]	8
Fan et al. (15)	China	RCS	2013–2017	mCRPC	AA-to-DP or DP-to-AA	104	72 [65.3–77.0]	200	OS/PFS	19.2 [17.3–22.3]	8
Lolli et al. (12)	Italy	RCS	2011.04–2015.05	mCRPC	D-to-AA	230	74 (45–90)	535	OS	29 (1–55)	8
Man and Chen (23)	China	RCS	2010–2018	mCRPC	D	179	70 (51–88)	535	OS	24 (2–118)	8
Kobayashi et al. (22)	Japan	RCS	2008–2018	mCRPC	D + ADT	144	71 [65–76]	636	OS/PFS	NR	7
Stangl-Kremser et al. (26)	Austria	RCS	2005–2016	mCRPC	D + ADT	186	68.8 [64.6–75.0]	200	OS/PFS	25.5 [12.8–42.4]	8

[ ], interquartile range; ( ), range; AA, abiraterone; ADT, androgen deprivation therapy; D, docetaxel; DP, docetaxel-prednisone; EZ, Enzalutamide; mCRPC, metastatic castration-resistant prostate cancer; NR, Not Reported; OS, overall survival; PCS, prospective cohort study; PFS, progression-free survival; [^223^Ra] RaCl_2_, radium-223 dichloride; RCS, retrospective cohort study.

More than 90% of patients included in studies conducted by Kobayashi et al. and Stangl-Kremser et al. were diagnosed with mCRPC and <10% were diagnosed with nmCRPC, so these studies were therefore assigned to the mCRPC group.

**Table 2:** Baseline characteristics of included nmPCa patients.

**References**	**Country**	**Study design**	**Study period**	**Tumor type**	**Treatment**	**Sample size**	**Age**	**Cutoff value**	**Outcome**	**Follow-up (months)**	**NOS SCORE**
Rajwa et al.* (24)	Multi-country	RCS	2007–2015	nmrPCa	SRP	214	69 [64–72]	730	BFS/MFS/CSS/OS	25.3 [15–28.5]	7
Rajwa et al. (25)	Multi-country	RCS	2000–2011	nmPCa	RP	6039	61 [57–66]	620	BFS	44 [31–57]	8
Wang et al. (27)	China	RCS	2014.01–2019.12	nmPCa	RP	291	66.13 ± 6.05	528.54	BFS	48 [36–62]	8

[ ], interquartile range; BFS, biochemical recurrence-free survival; CSS, cancer-specific survival; MFS, metastasis-free survival; nmPCa, non-metastatic prostate cancer; nmrPCa, non-metastatic radio-recurrent prostate cancer; OS, overall survival; RCS, retrospective cohort study; RP, radical prostatectomy; SRP, salvage radical prostatectomy. *Two studies were reported by Rajwa et al. with different populations.

### Quality assessment

The outcomes of quality assessment are shown in Tables 1, 2. The NOS score of each study was equal to or higher than 7, which suggested that all studies were of acceptable quality.

### Association between the SII and OS in mCRPC patients and nmPCa patients

**Figure 2:**
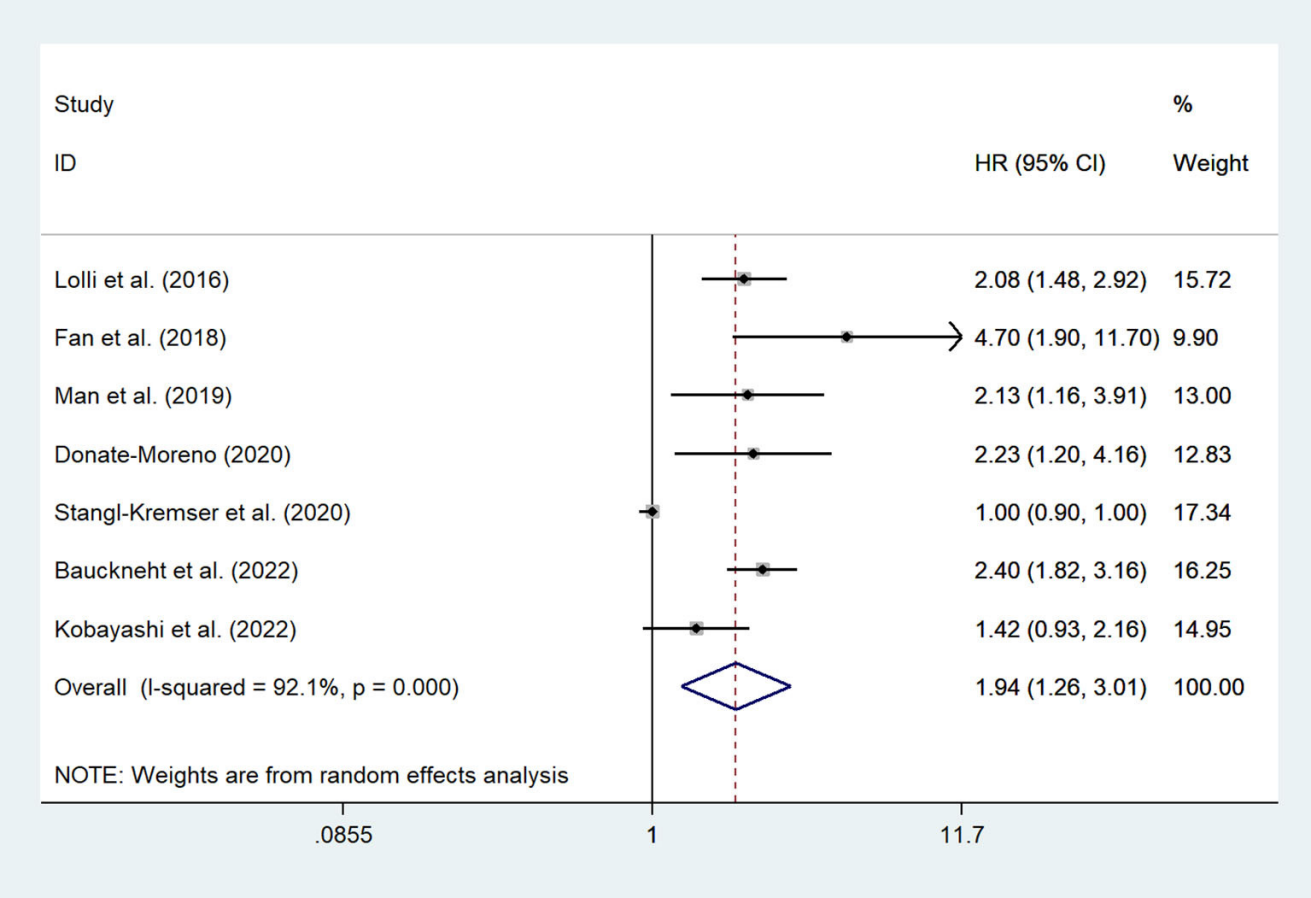
Forest plot of the association between SII and OS in mCRPC patients. SII, systemic immune-inflammation index; OS, overall survival; mCRPC, metastatic castration-resistant prostate cancer.

There are 7 studies (12, 15, 20–23, 26) involving mCRPC patients who reported the association between the SII and OS. The pooled analysis indicated that the mCRPC patients with high SII had a significantly worse OS (HR = 1.94, 95% CI: 1.26–3.01, *p* = 0.003), with significant heterogeneity between studies (*I*^2^ = 92.1%, *p* < 0.001), as shown in Figure 2. Besides, one study (25) reported the association between the SII and OS in nmrPCa patients, and it indicated that patients with high SII had a significantly worse OS (HR = 8.57, 95% CI: 2.70–27.2, *p* < 0.001).

### Association between the SII and PFS in mCRPC patients

**Figure 3:**
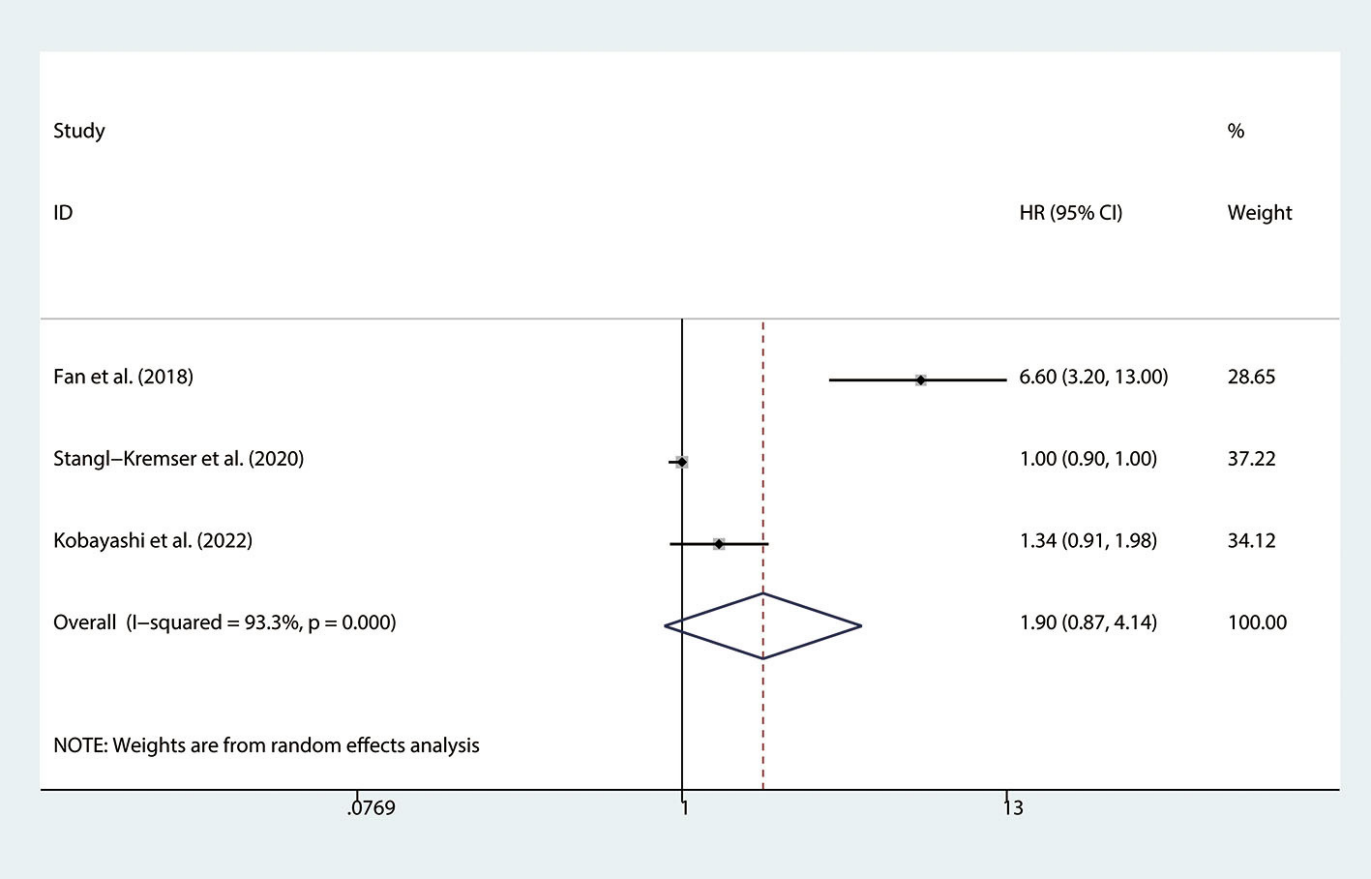
Forest plot of the association between SII and PFS in mCRPC patients. SII, systemic immune-inflammation index; PFS, progression-free survival; mCRPC, metastatic castration-resistant prostate cancer.

There are 3 studies (15, 22, 26) involving mCRPC patients who reported the association between the SII and PFS. We found that there was no significant association observed between SII and PFS (HR = 1.90, 95% CI: 0.87–4.14, *p* = 0.107), with significant heterogeneity between studies (*I*^2^ = 93.3%, *p* < 0.001), as shown in Figure 3.

### Association between the SII and BFS in nmPCa patients

**Figure 4:**
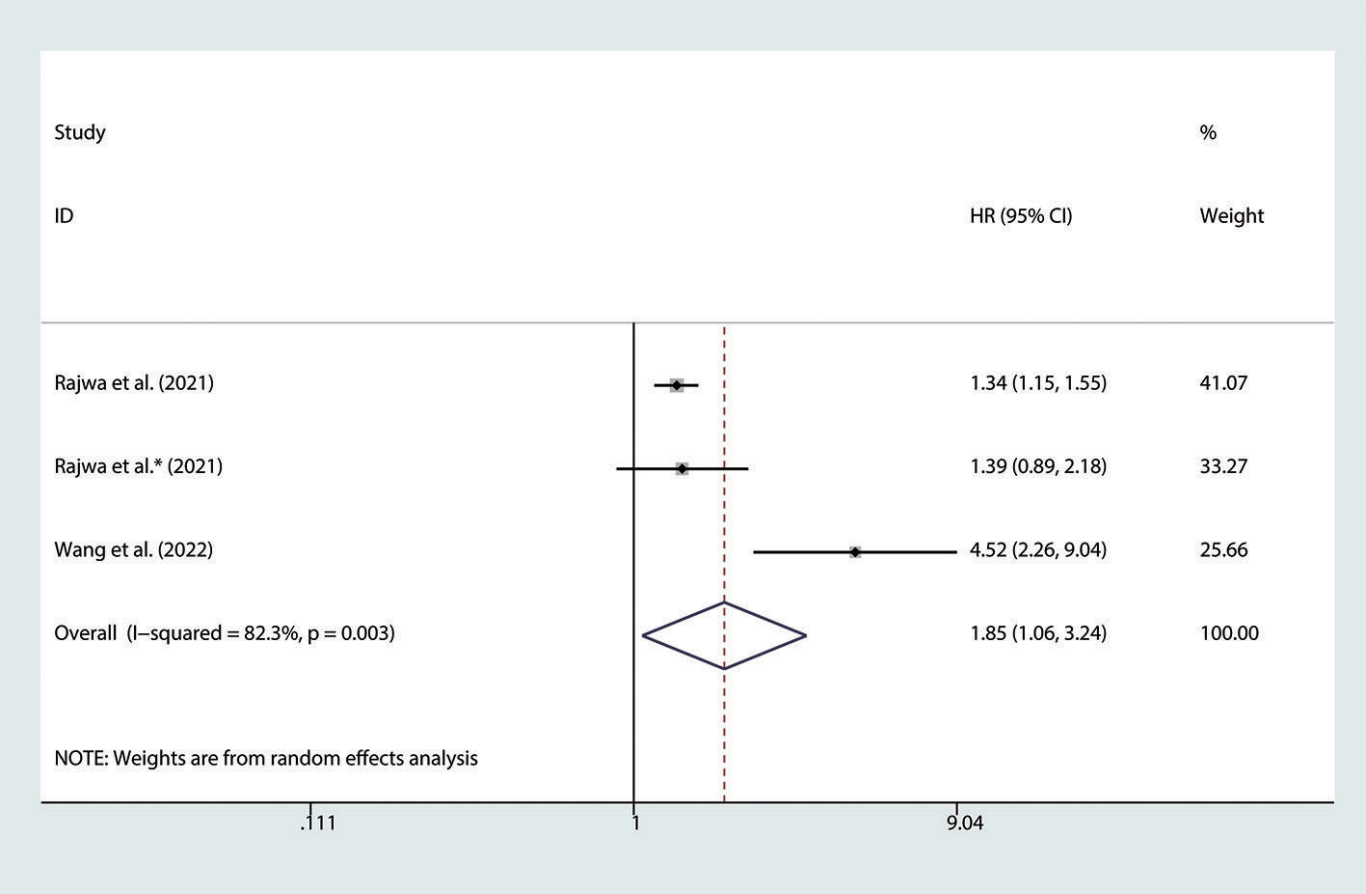
Forest plot of the association between SII and BFS in nmPCa patients with prostate cancer. SII, systemic immune-inflammation index; BFS, biochemical recurrence-free survival; nmPCa, non-metastatic prostate cancer.

There are 3 studies (24, 25, 27) involving nmPCa patients who reported the association between the SII and BFS. The pooled analysis indicated that nmPCa patients with high SII had a significantly worse BFS (HR = 1.85, 95% CI: 1.06–3.24, *p* = 0.031), with significant heterogeneity between studies (*I*^2^ = 82.3%, *p* = 0.003), as shown in Figure 4.

### Association between the SII and clinicopathological factors in nmPCa patients

**Figure 5:**
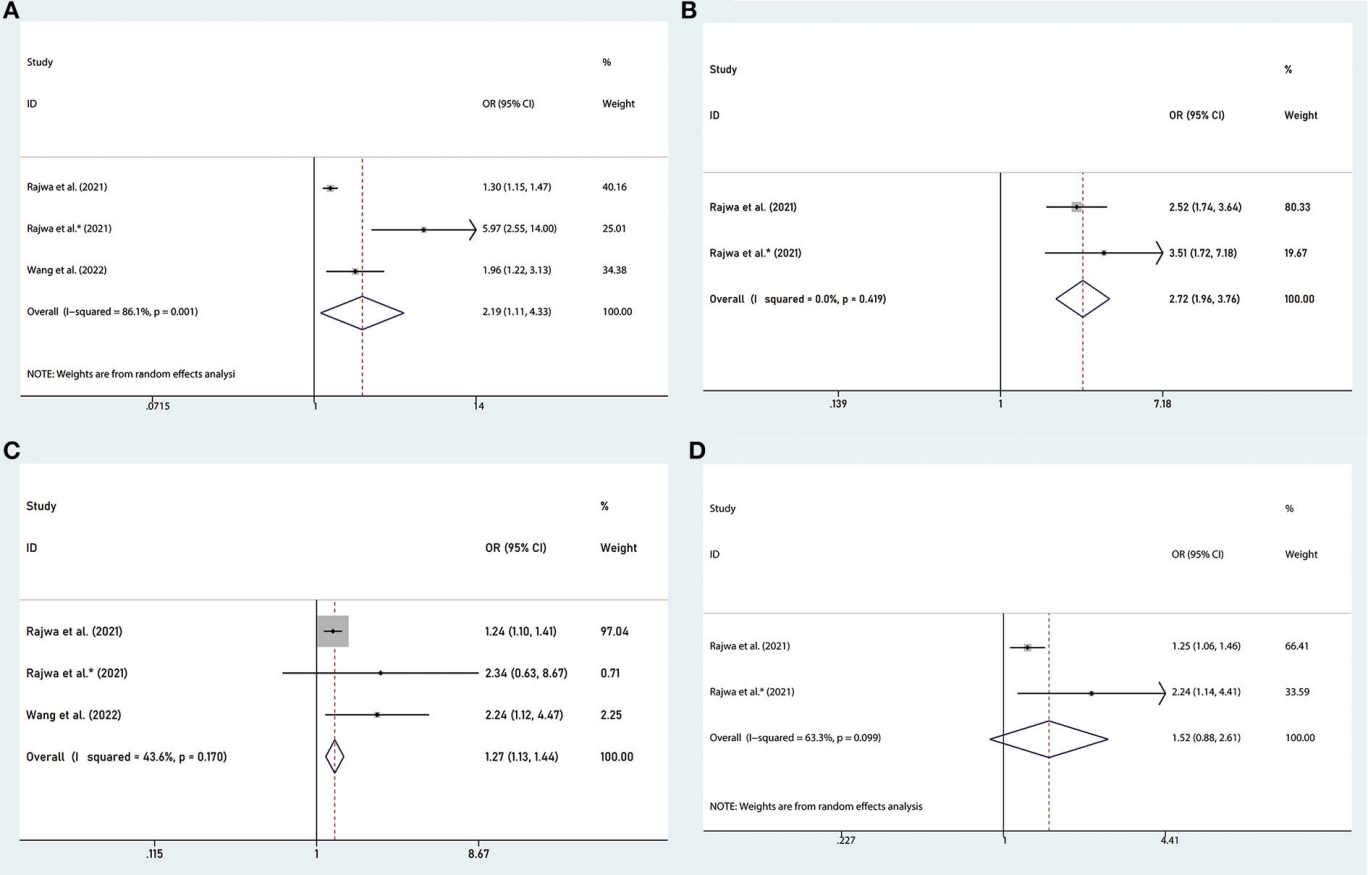
Forest plots of the association between SII and adverse clinicopathology in nmPCa: **(A)** pathological tumor stage ≥3, **(B)** pathological lymph node stage ≥1, **(C)** Gleason score ≥7, and **(D)** positive surgical margins. SII, systemic immune-inflammation index; nmPCa, non-metastatic prostate cancer.

RP is the preferred treatment for patients with nmPCa. And the clinicopathological characteristics of surgical specimens are important indicators of the prognosis of patients. Several studies reported the association between the SII and adverse clinicopathology (24, 25, 27), including tumor stage (pT ≥3 vs. pT <3), lymph node involvement (pN ≥1 vs. pN = 0), Gleason score (GS ≥7 vs. GS <7), the status of surgical margins (positive vs. negative). We found that the high SII was associated with advanced tumor stage of PCa (OR = 2.19, 95% CI: 1.11–4.33, *p* = 0.024), presence of lymph node involvement (OR = 2.72, 95% CI: 1.96–3.76, *p* < 0.001) and Gleason score (OR = 1.27, 95% CI: 1.13–1.44, *p* < 0.001), but we found there was no significant association between SII and the status of surgical margins (OR = 1.52, 95% CI: 0.88–2.61, *p* = 0.132), as shown in Figure 5.

### Subgroup analysis

**Figure 6:**
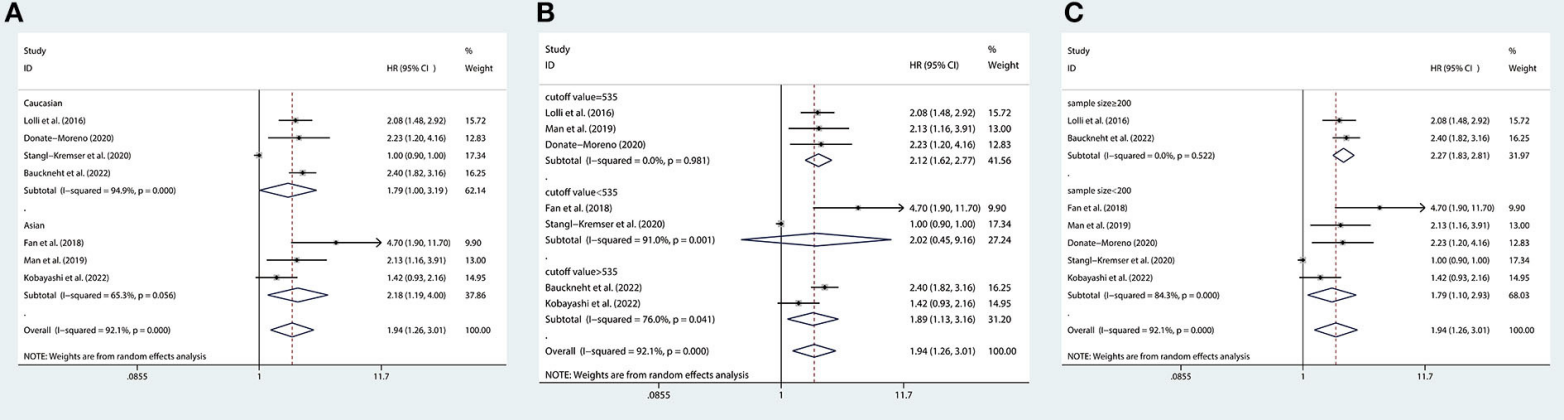
Subgroup analyses of OS according to **(A)** ethnicity, **(B)** cutoff value, and **(C)** sample size. OS, overall survival.

We performed subgroup analyses to further assess the association between SII and OS in mCRPC patients. Subgroup analyses were conducted by ethnicity, cutoff value, and sample size. There are 3 studies with a cutoff value of 535, and there are 2 studies with a cutoff value <535 and 2 studies with a cutoff value >535, so we divided all studies into three subgroups based on the cutoff value of 535.The results of the subgroup analysis showed that a higher SII was not significantly associated with worse OS in patients of the subgroup with a cutoff value <535, but it was significantly associated with worse OS in patients of the subgroup with a cutoff value >535 or =535. In addition, we found that a higher SII was associated with worse OS in patients regardless of ethnicity and sample size (Figure 6).

### Results of qualitative analysis

Neuberger et al. studied the predicting significance of SII and other indicators for the prognosis of patients with metastatic hormone-sensitive prostate cancer, and they found that there was no significant role for SII in predicting therapy response or prognosis (16). Although this study was excluded from the meta-analysis because it didn't provide the specific value of HR and 95% CI, it is unlikely to change our conclusion because its sample size is too small.

### Sensitivity analysis

**Figure 7:**
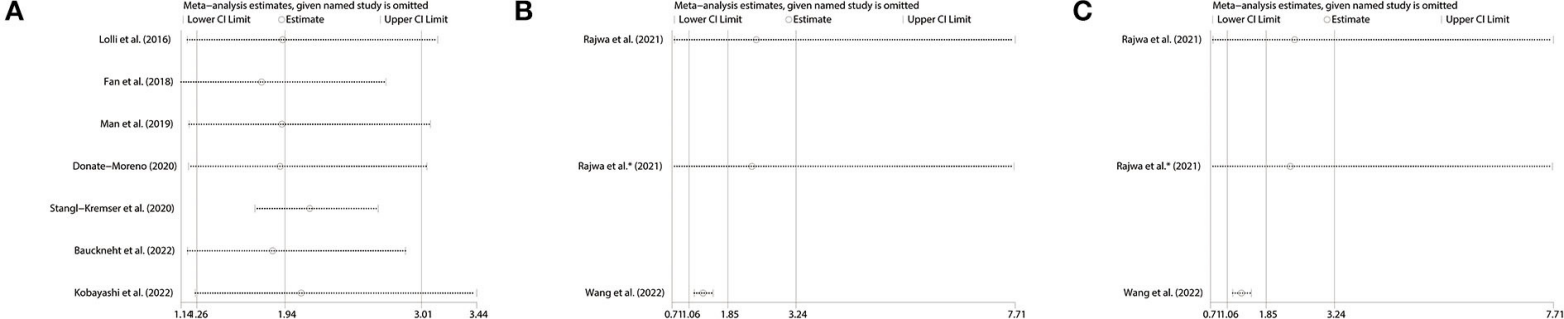
Sensitivity analysis of pooled results for **(A)** OS, **(B)** PFS in mCRPC patients and **(C)** BFS in nmPCa patients. OS, overall survival; PFS, progression-free survival; BFS, biochemical recurrence-free survival; mCRPC, metastatic castration-resistant prostate cancer; nmPCa, non-metastatic prostate cancer.

We conducted sensitivity analyses by omitting individual studies sequentially to assess the reliability of pooled HRs for OS, PFS and BFS. We found there was no significant change in the overall HR estimates for these survival outcomes, which suggested that the results of our meta-analysis were stable, as shown in Figure 7.

## Discussion

PCa is a malignant tumor with a high incidence rate, which is a serious health hazard for males (1). At present, the main predictors for the prognosis of patients with PCa are clinicopathological indicators, like pT stage, GS and PSA level, and hematological indicators can serve as complementary predictors. SII is an index based on the counts of neutrophils, lymphocytes and platelets, which can reflect the inflammatory microenvironment of the body. To our knowledge, this review is the first to specifically explore the association between high SII and prognosis in patients with different stage PCa. We finally included 10 studies which contained a total of 7,986 patients in our meta-analysis, 1,442 patients in 7 studies (12, 15, 20–23, 26) were diagnosed with mCRPC, and 6,544 patients in the other 3 studies (24, 25, 27) were diagnosed with nmPCa. Because PCa is a complex and heterogenous disease, we divided the included studies into different groups according to the stage. We found that elevated SII was associated with worse OS and BFS, but not worse PFS. In addition, elevated SII was also associated with higher tumor stage (pT ≥ 3) Gleason score (GS ≥ 7) and lymph node involvement (pN ≥ 1). Elevated SII was not significantly associated with worse OS in the subgroup with a cutoff value of SII <535, other subgroup analyses did not alter the direction of results for OS. Therefore, we believe that elevated SII is associated with poorer prognosis in PCa patients.

The relationship between elevated SII and prognosis in patients with malignant urologic tumors has been controversial for a long time. Huang et al. conducted a meta-analysis including 14 studies and 3,074 patients with urologic tumors (28), and there are three studies of patients with PCa in this study. They found that elevated SII was associated with poorer OS, PFS and cancer-specific survival (CSS) in patients with urinary system tumors. Besides, the results of PCa subgroup analysis showed that elevated SII was associated with bad prognosis in PCa patients. Wang et al. conducted a meta-analysis including 12 studies and 2,693 patients with urinary system tumors and they reached similar conclusions (29). The PCa patients included in these two meta-analyses were mainly diagnosed with mCRPC, and the treatment for patients was mainly docetaxel-based chemotherapy. Our study included several newly published studies (20–22, 24, 25, 27), and patients included in 3 studies (24, 25, 27) were pathologically diagnosed with nmPCa and the treatment for these patients was surgical treatment, which was different from previous studies.

There were also many studies exploring the relationships between elevated SII and the prognosis of patients with other malignant tumors, such as pancreatic cancer, liver cancer, small cell lung cancer, gynecological tumors, and breast cancer (30–32). Based on the results of these studies, it appears that patients with an elevated SII had a poor prognosis. However, in all studies, a determined cutoff value of SII has not yet been defined. Therefore, we think that determining the appropriate cutoff values for different diseases or patients will be the focus of future work.

In addition to SII, other inflammatory markers have also been widely explored for their predictive role in the prognosis of PCa patients. Guo et al. investigated the prognostic value of the NLR and PLR in PCa (33). They found that elevated pretreatment NLR was associated with poor OS, PFS and BFS, and high pretreatment PLR was correlated with inferior PFS, OS and CSS. Guan et al. focused on mCRPC patients who were treated with abiraterone or enzalutamide, and they found that NLR and PLR were effective biomarkers for predicting prognosis in mCRPC patients treated with abiraterone or enzalutamide (10).

The mechanism underlying the prognostic value of inflammatory factors such as SII, NLR and PLR for PCa patients is still unclear, the most accepted opinion is that it is related to changes in the tumor microenvironment (34). During tumorigenesis, neutrophils are involved in the process of tumor cell proliferation, invasion and migration, and tumor immunosuppression (35, 36). The increase of neutrophils promotes the release of inflammatory factors such as vascular epithelial growth factor (VEGF), interleukin-8 (IL-8), IL-16, and so on (37). These cytokines produced in the tumor microenvironment promote tumorigenesis and progression. Patients with cancer often suffer from a hypercoagulable state (38), and platelets can increase blood coagulation ability to cause hypercoagulability. On the other hand, platelet-derived tumor growth factor-beta (PDGF-β) can protect tumor cells from immune system surveillance (29). Moreover, once platelets accumulate in peripheral blood, they can stimulate tumor angiogenesis and promote tumor growth (39). Lymphocytes are the major cells involved in cell-mediated immunity, and they also participate in cancer immune surveillance and immune editing. Lymphocytes can promote cytotoxic cell death and inhibit tumor cell proliferation and migration, thereby playing a crucial role in tumor defense (40). Decreased lymphocytes may lead to cancer cells escaping immune surveillance and causing the development of malignancies. Therefore, changes in these peripheral blood cells may imply changes in the human tumor microenvironment, thereby affecting the prognosis of patients.

In addition to the predictive role in prognosis, Sonmez et al. found that SII also plays a role in the diagnosis of PCa. SII had significant diagnostic value in PCa patients with an ISUP grade ≥3, the combination of SII with the PI-RADS score might be the most effective marker to diagnose PCa (41). Wang et al. also found that patients with PCa had significantly higher SII than those diagnosed with benign prostatic hyperplasia (42). It provides more possibilities for the application of SII in clinical practice.

There are still some limitations in our study. Firstly, only one study included was prospective cohort study. Secondly, there is only one study with a large sample size, and the other studies have small sample sizes. Thirdly, the cutoff values of the included studies are not completely consistent, and the results of subgroup analysis showed that a higher SII was not significantly associated with a worse OS in mCRPC patients of the subgroup with a cutoff value <535, this indicated that determining the appropriate cutoff value will be the focus of future work. Fourthly, if the number of included studies was <10, publication bias couldn't be performed (43, 44). The pooled results of OS, PFS and BFS included 7, 3, and 3 studies, respectively, so we couldn't study the publication bias. It means that there may be publication bias in our studies, this is one of the main shortcomings of our study, and it may be the source of heterogeneity, so we need more studies to further confirm our findings. We hope that there will be more prospective studies with large sample sizes in the future to validate our conclusions.

## Conclusions

High SII was associated with bad OS in mCRPC patients, and associated with bad BFS and adverse pathological features in nmPCa patients. We think SII can be a prognostic predictor for PCa patients. The application of SII will advance the diagnosis and treatment of prostate cancer.

## Data availability statement

The raw data supporting the conclusions of this article will be made available by the authors, without undue reservation.

## Author contributions

WQ, YHZ, and YFZ: conception and design. BS and YFZ: administrative support. WQ, YHZ, ZL, and JW: provision of study materials or patients. WQ, YHZ, GL, and MZ: collection and assembly of data. WQ and YHZ: data analysis and interpretation. All authors contributed to the manuscript writing and final approval of manuscript.

## Funding

This work was supported by the Natural Science Foundation of Shandong Province (ZR2021MH318 to YFZ).

## Conflict of interest

The authors declare that the research was conducted in the absence of any commercial or financial relationships that could be construed as a potential conflict of interest.

## Publisher's note

All claims expressed in this article are solely those of the authors and do not necessarily represent those of their affiliated organizations, or those of the publisher, the editors and the reviewers. Any product that may be evaluated in this article, or claim that may be made by its manufacturer, is not guaranteed or endorsed by the publisher.
