# Supplementary material for: Revealing the prognostic and clinicopathological significance of systemic immune-inflammation index in patients with different stage prostate cancer: A systematic review and meta-analysis
Source: Front Med (Lausanne). 2022 Oct 31;9:1052943. doi: 10.3389/fmed.2022.1052943 (PMC9659961; doi:10.3389/fmed.2022.1052943)
Supplement: Supplementary file 1 [file Table_1.DOCX]

**Table S1** Details of search strategy for all databases

| **Database** | **Search Strategy** |
| --- | --- |
| **PubMed** | (((((((((((((((((Prostate Neoplasms) OR (Neoplasms, Prostate)) OR (Neoplasm, Prostate)) OR (Prostate Neoplasm)) OR (Neoplasms, Prostatic)) OR (Neoplasm, Prostatic)) OR (Prostatic Neoplasm)) OR (Prostate Cancer)) OR (Cancer, Prostate)) OR (Cancers, Prostate)) OR (Prostate Cancers)) OR (Cancer of the Prostate)) OR (Prostatic Cancer)) OR (Cancer, Prostatic)) OR (Cancers, Prostatic)) OR (Prostatic Cancers)) OR (Cancer of Prostate)) AND (((((systemic immune-inflammation index) OR (systemic-immune-inflammation index)) OR (neutrophil × platelets/lymphocyte)) OR (platelet count × NLR)) OR (SII)) |
|  |  |
| **Embase** | ('prostate cancer'/exp OR 'cancer, prostate' OR 'malignant prostate tumor' OR 'malignant prostate tumour' OR 'malignant prostatic tumor' OR 'malignant prostatic tumour' OR 'prostate gland cancer' OR 'prostate malignancy' OR 'prostate malignant neoplasm' OR 'prostate malignant tumor' OR 'prostate malignant tumour' OR 'prostatic cancer' OR 'prostatic malignancy') AND ('systemic immune inflammation index'/exp AND 'systemic immune-inflammation index' OR 'systemic-immune-inflammation index' OR 'neutrophil × platelets/lymphocyte' OR 'platelet count × nlr' OR 'sii') |
|  |  |
| **Cochrane Library** | ((Prostatic Neoplasms [MeSH]) OR ((Neoplasm, Prostatic) OR (Neoplasm, Prostate) OR (Neoplasms, Prostate) OR (Prostate Neoplasm) OR (Neoplasms, Prostatic) OR (Prostatic Neoplasm) OR (Prostate Neoplasms) OR (Cancers, Prostate) OR (Prostatic Cancer) OR (Prostate Cancers) OR (Prostatic Cancers) OR (Prostate Cancer) OR (Cancer, Prostatic) OR (Cancers, Prostatic) OR (Cancer, Prostate) OR (Cancer of Prostate) OR (Cancer of the Prostate))) AND ((systemic immune-inflammation index) OR (systemic-immune-inflammation index) OR (SII)) |
